# Revealing the Hazard of Mild Electrical Abuse on the Safety Characteristics of NaNi_1/3_Fe_1/3_Mn_1/3_O_2_ Cathode Sodium‐Ion Battery

**DOI:** 10.1002/advs.202501649

**Published:** 2025-04-25

**Authors:** Qinghua Gui, Bowen Jin, Peng Liu, Kun Yu, Jiarui Zhang, Lei Mao

**Affiliations:** ^1^ Department of Precision Machinery and Precision Instrumentation University of Science and Technology of China Hefei 230027 China; ^2^ Institute of Advanced Technology University of Science and Technology of China Hefei 230031 China

**Keywords:** capacity degradation, mild electrical abuse, postmortem characterization analysis, sodium‐ion battery, thermal characteristic

## Abstract

This study investigates the degradation of safety characteristics in commercial NaNi_1/3_Fe_1/3_Mn_1/3_O_2_ (NFM) sodium‐ion batteries (SIBs) under mild electrical abuse, focusing on the impact of a single electrical abuse event on capacity, impedance characteristics, and thermal stability, and reveals the degradation pattern of safety characteristics under long‐term electrical abuse cycles. The research finds that while a single mild electrical abuse event has a limited effect on battery capacity, the internal electrochemical properties of the battery still change. Mild overcharge reduces the thermal stability of the anode material, and the increased sodium plating on the anode surface also significantly affects the overall safety characteristics of the battery. In contrast, the harm caused by over‐discharge is less severe. After long‐term electrical abuse cycles, the battery experiences significant capacity degradation, and prolonged overcharge cycles cause further sodium plating, severely affecting battery safety. Overall, the impact of long‐term mild overcharge on safety characteristics is more pronounced. This study not only clarifies the degradation mechanism of sodium‐ion battery safety characteristics under electrical abuse but also provides theoretical support for the optimization design and safety evaluation of sodium‐ion batteries.

## Graphical abstract

The mechanism of capacity degradation of NaNi_1/3_Fe_1/3_Mn_1/3_O_2_ (SIBs) under mild electric abuse is revealed. The effect of mild electrical abuse on the thermal characteristics of SIBs is discussed. The degradation path of SIBs under long‐term mild electrical abuse is clarified. Elucidate the coupling mechanisms between electrode materials, electrochemical signals, and heat generation characteristics.

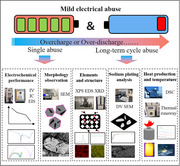

## 1 Introduction

With the rapid development of renewable energy and electric transportation, battery technology has become a cornerstone in the field of energy storage.^[^ ^1^ ^]^ Sodium‐ion batteries (SIBs) have garnered increasing attention as a potential alternative to lithium‐ion batteries (LIBs) due to their abundant resources, low cost, and relatively high safety.^[^ ^2^, ^3^ ^]^ While sodium‐ion batteries exhibit slightly lower energy density compared to lithium‐ion batteries, they show promising prospects for large‐scale energy storage systems and low‐cost applications like two‐wheeled electric vehicles.^[^ ^4^ ^]^ However, the safety of SIBs remains the critical issue that needs to be addressed during their commercialization process,^[^ ^5^ ^]^ particularly in terms of performance degradation and thermal runaway behavior under abusive conditions such as overcharge, over‐discharge, and short circuits. Since mild electrical abuse may be experienced in practical applications, it is of significant theoretical and practical importance to investigate its impact on the safety characteristics of SIBs.

In practical operation, battery packs are typically configured in series or parallel arrangements. Due to inconsistencies among individual cells within the pack, such as manufacturing defects, uneven aging, or improper calibration of the Battery management system (BMS), overcharge or over‐discharge may occur,^[^ ^6^ ^]^ while mild overcharge and over‐discharge are often difficult for the BMS to detect efficiently. Prolonged exposure to electrical abuse not only accelerates the degradation of electrode materials, leading to irreversible capacity loss,^[^ ^7^, ^8^ ^]^ but also impacts the safety characteristics of the battery materials. In extreme cases, this may even trigger thermal runaway (TR) and result in catastrophic accidents such as fires or explosions.^[^ ^9^ ^]^

Research on the overcharge behavior of LIBs has been relatively extensive. Overcharge is typically believed to lead to gas generation within the battery, and when the internal pressure from the gas expansion exceeds a critical limit, it may result in a battery explosion. Ren et al.^[^ ^10^ ^]^ studied commercial pouch LIBs and found that severe overcharge caused electrolyte decomposition, transition metal dissolution, and phase transitions in the positive electrode, while the deposition of metal on the negative electrode could potentially trigger an Internal short circuit (ISC), posing a safety risk.^[^ ^11^, ^12^ ^]^ Zhang et al.^[^ ^13^ ^]^ conducted research on 18650 LIBs and found that although a single mild electrical abuse event would not cause significant capacity degradation, mild overcharge‐induced lithium plating and mild over‐discharge‐induced copper plating were the key factors responsible for the change in the thermal characteristics of LIBs. Xu et al.^[^ ^14^ ^]^ used overcharge conditions to induce TR in LIBs and discovered that early swelling is caused by lithium plating, and CO generated from incomplete oxidation of the electrolyte was the primary cause of the rapid swelling. Compared to LIBs, research on SIBs is still in its early stages. However, previous studies have shown that the degradation mechanism of SIBs under overcharge conditions is similar to that of LIBs, while larger size and lower diffusion rate of sodium ions make SIBs more susceptible to severe electrode damage under overcharge conditions. Gui et al.^[^ ^15^ ^]^ conducted overcharge studies on sodium‐ion full cells with two different material systems, NaNi_1/3_Fe_1/3_Mn_1/3_O_2_ and Na_4_Fe_3_(PO_4_)_2_(P_2_O_7_) and found that overcharge accelerated side reactions, gas generation, and sodium plating, causing rapid swelling of the battery and an increase in temperature until TR is triggered. It is evident that electrical abuse poses a significant threat to both LIBs and SIBs.

As another critical form of battery abuse, over‐discharge also poses significant hazards to battery capacity and safety characteristics. For LIBs, existing studies indicate that over‐discharge often leads to the dissolution of the anode current collector, where dissolved copper ions redeposit on the electrode surface to form copper dendrites. Similar to lithium dendrites, these copper dendrites can puncture the separator, causing ISC.^[^ ^16^, ^17^ ^]^ Additionally, over‐discharge damages the solid electrolyte interphase (SEI) layer and causes sustained decomposition of the electrolyte. The byproducts generated not only increase the internal resistance of the battery but also accelerate irreversible capacity decay.^[^ ^18^ ^]^ Ma et al. experimentally demonstrated that over‐discharge significantly reduces the structural stability of LIB electrode materials, a degradation process accompanied by substantial heat release, further increasing the risk of TR.^[^ ^19^ ^]^ Sethuraman et al. revealed that the irreversible copper deposition and byproduct formation during over‐discharge severely hinder the normal migration of lithium ions, leading to a significant reduction in the cycle life of the battery.^[^ ^20^ ^]^ Beyond capacity degradation, over‐discharge can also exacerbate TR behavior. Studies have shown that over‐discharge promotes rapid decomposition of the electrolyte, releasing flammable gases such as CO and CH_4_. The accumulation of these gases dramatically increases internal pressure within the battery, potentially causing catastrophic incidents such as explosions.^[^ ^21^, ^22^, ^23^, ^24^ ^]^

Although SIBs share similarities with LIBs in terms of electrochemical energy storage principles and electrode material degradation mechanisms, research on SIBs under abusive conditions at the full‐cell level still remains limited.^[^ ^25^, ^26^, ^27^ ^]^ Most existing studies have focused on the thermal stability, electrochemical behavior, and interfacial properties of individual electrode materials, while investigations into the performance evolution, degradation mechanisms, and safety evaluation of full SIBs under complex operating conditions are still in their infancy.^[^ ^5^ ^]^ Due to the larger ionic radius and lower diffusion rate of sodium ions, these characteristics may influence the degradation rate and failure modes of SIBs under extreme conditions.^[^ ^28^, ^29^ ^]^ As a result, the mild overcharge and over‐discharge behaviors of SIBs may exhibit distinct characteristics compared to LIBs,^[^ ^30^ ^]^ necessitating further experimental validation and analysis.

Based on the literature review, numerous studies have been conducted on SIBs with various cathode material systems, including layered oxides, polyanionic compounds, and Prussian blue analogs.^[^ ^31^, ^32^ ^]^ Research on SIBs layered oxides is primarily focused on the O3 and P2 phases, with the general formula for layered oxide cathodes being NaxTMO_2_, with multiple variations depending on the doping elements and their ratios.^[^ ^33^ ^]^ Among these, O3‐type layered oxide materials, such as NaNi_1/3_Mn_1/3_Fe_1/3_O_2_ (NFM) stand out due to their high specific capacity, low cost, and environmental friendliness, making them one of the most promising cathode materials for SIBs.^[^ ^34^ ^]^ However, limited research has focused on the safety characteristics of NaNi_1/3_Mn_1/3_Fe_1/3_O_2_ under full‐cell operating conditions. This is particularly critical for large‐scale energy storage systems, where long‐term cycling often results in mild electrical abuse of individual cells. Therefore, it is imperative to investigate the degradation mechanisms and pathways of safety characteristics under single and long‐term mild electrical abuse for SIBs.

Based on existing reports on LIBs and our research on SIBs, it is found that under a single overcharge not exceeding 120% SOC or a single over‐discharge not exceeding 110% SOC, the battery will not directly trigger TR or cause catastrophic accidents, and the battery will not show obvious physical deformation, swelling or leakage.^[^ ^10^, ^14^ ^]^ However, such charging above the rated upper voltage of the battery and discharge above the rated lower voltage may accelerate the degradation of the battery. Therefore, this degree of overcharge and over‐discharge is defined as mild electrical abuse.

This study investigates the degradation mechanisms and pathways of safety characteristics of SIBs under mild electrical abuse. Initially, the electrochemical performance and sodium plating behavior are examined using incremental capacity (IC) tests, electrochemical impedance spectroscopy (EIS), and differential voltage (DV) curves from relaxation voltage. In addition, overcharge‐induced TR experiments are conducted to assess the impact of mild electrical abuse on TR trigger temperature and onset. In order to further understand the relationship between macro phenomena and micro changes, the morphology, elemental composition, and phase structure of the abused electrodes are characterized using scanning electron microscopy (SEM), energy‐dispersive spectroscopy (EDS), X‐ray photoelectron spectroscopy (XPS), and X‐ray diffraction (XRD). Furthermore, differential scanning calorimetry (DSC) is used to analyze heat generation characteristics. Finally, long‐term cycling tests reveal the degradation pathways of electrochemical and safety characteristics under prolonged mild electrical abuse. The results can provide theoretical insights and experimental support for the safety design and failure mechanism research of SIBs.

## 2 Results and Discussion

### 2.1 Effect of Electrical Abuse on Electrochemical Performance

**Figure 1:**
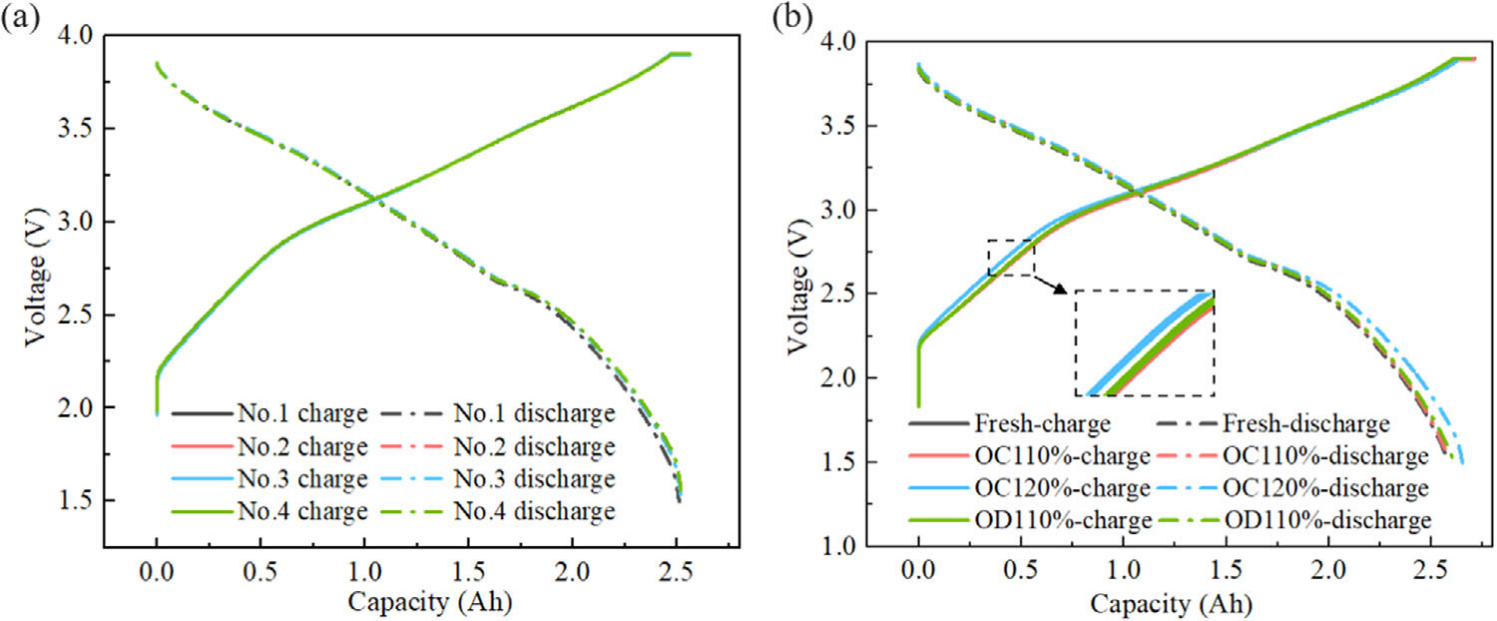
Comparison of charge and discharge curves before and after electrical abuse. a) Before abuse. b) After abuse.

**Figure** **1**a shows standard charge‐discharge curves of four NFM SIBs under constant current‐constant voltage (CC‐CV) charge and constant current discharge (CCD) at 1C‐rate. The curves of the four batteries overlap almost perfectly without deviation, and the voltage plateau remains stable. This indicates the reliability of the battery manufacturing process, as well as the uniform distribution of internal resistance and active electrode materials. Similarly, discharge capacities also demonstrate a high degree of consistency.

Figure 1b presents charge‐discharge curves after suffering 110% SOC and 120% SOC overcharge, as well as −110% SOC over‐discharge. The results show that mild electrical abuse has little effect on voltage curves during cycling, and only slightly higher initial voltage compared to fresh batteries is observed after 120% SOC overcharge. Moreover, after a single instance of minor electrical abuse, battery capacity even shows a slight increase. This may be attributed to the surplus capacity designed into the battery, where sodium‐ion intercalation or de‐intercalation sites that originally did not participate in the reaction are activated, resulting in slight short‐term increase in capacity.

To further investigate the impact of mild electrical abuse on the electrochemical performance degradation of SIBs, IC tests are conducted to reveal internal phase transition behaviors, electrode reaction pathways, and the evolution of active materials. EIS tests are employed to assess the extent of interface and transport process deterioration before and after electrical abuse by measuring changes in internal impedance. Additionally, relaxation voltage tests, which reflect battery polarization changes, sodium‐ion distribution uniformity, and internal stress release, are performed.^[^ ^35^, ^36^, ^37^ ^]^

**Figure 2:**
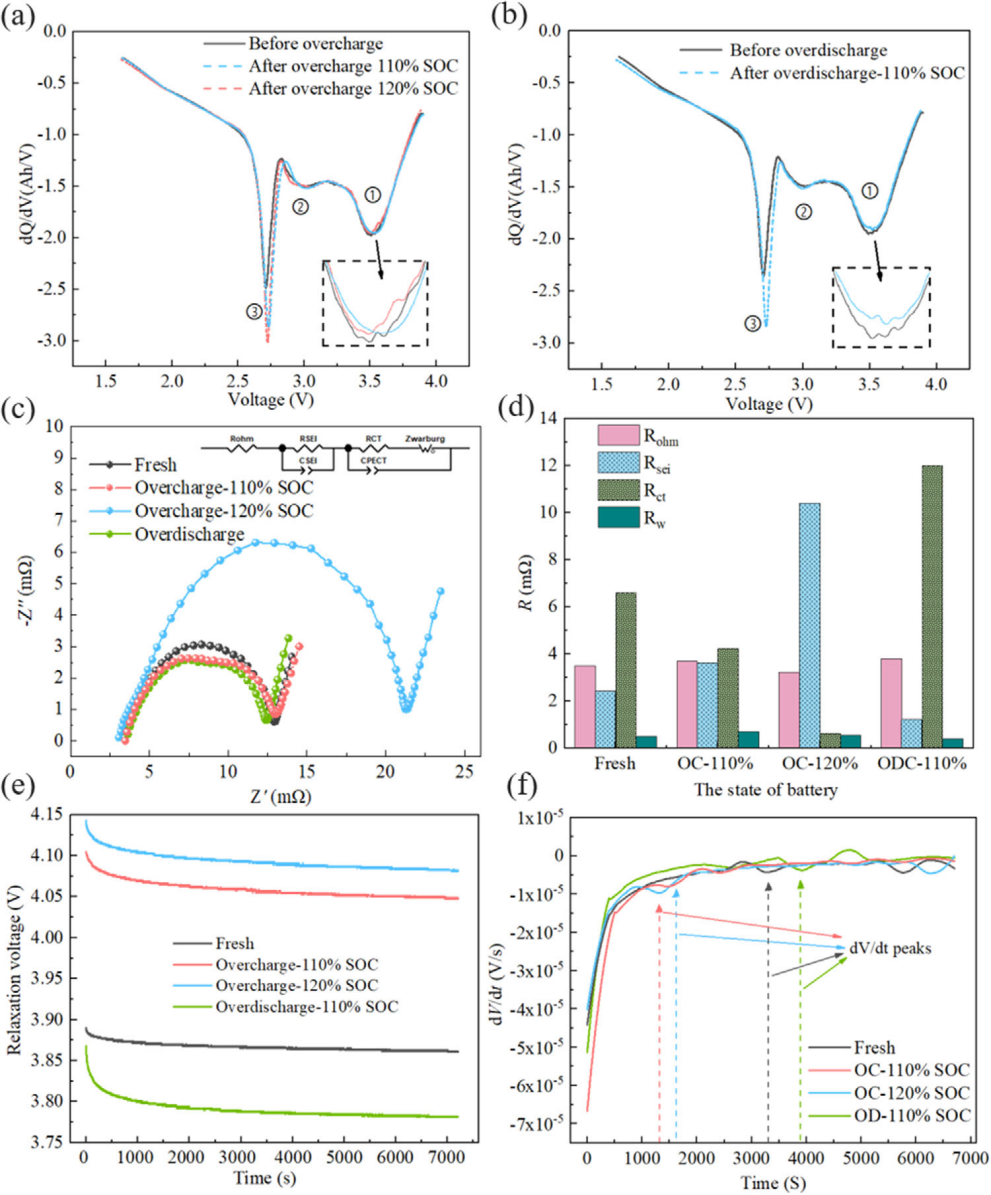
Degradation of electrochemical performance of SIBs after electrical abuse. a) Comparison of IC curve changes caused by overcharge. b) Comparison of IC curve changes caused by over‐discharge. c) Comparison of EIS curve changes caused by electrical abuse. d) The fitted impedance value changes. e) Comparison of relaxation curve variation caused by electrical abuse. f) Comparison of DV curve variation based on relaxation voltage.

The detailed results are depicted in **Figure** **2**, and Figure 2a compares IC curves of fresh NFM SIB and those that suffered to 110% SOC and 120% SOC overcharge. It is evident that the discharge process of batteries features three characteristic peaks, and the overall voltage plateau remains unchanged, indicating that intercalation and de‐intercalation pathways of sodium ions are not disrupted, nor is the conductivity affected after mild overcharge. More specifically, a slight fluctuation in peak 1 suggests minimal impact on active material loss due to overcharge. The increased amplitude and area of peaks 2 and 3 in the 2.5–3.0 V range imply an increase in sodium inventory, attributed to the enhanced sodium ion migration in electrode materials during overcharge. Mild overcharge activates more sodium ions, allowing them to participate in migration. Figure 2b shows that IC curve changes for batteries subjected to mild over‐discharge follow a similar trend to those under mild overcharge. It should be noted that a slight decrease in the amplitude of peak 1 may be related to active material loss caused by over‐discharge, reflecting certain level of suppression of battery's kinetic performance at the high‐voltage region. Meanwhile, increased amplitude of peak 3 is consistent with behavior observed during overcharge. It is worth mentioning that the voltage measurement accuracy of the equipment is 0.02V. The measured peak deviation exceeds 0.06 V, with a relative error below 0.05%. Therefore, the peak deviation can be ensured to result from actual battery changes after mild electrical abuse rather than measurement error.

Figure 2c shows EIS test results for the four batteries after mild electrical abuse. The Nyquist curves can be divided into three regions, the high‐frequency region represents battery Ohmic resistance, the mid‐frequency region represents electrode‐electrolyte interface resistance and charge transfer resistance, and the low‐frequency region corresponds to sodium‐ion diffusion resistance. Compared to fresh batteries, semicircle arcs in Nyquist curves of 110% SOC overcharge and over‐discharge batteries shrink, while semicircle arc of 120% SOC overcharge batteries increases significantly. To further analyze the impact of electrical abuse on the impedance of various components, an equivalent circuit diagram is constructed as in Figure 2c for EIS curve fitting, and fitting results for 𝑅_ohm_ (ohmic resistance), 𝑅_sei_ (SEI film resistance), 𝑅_ct_ (charge transfer resistance), and 𝑅_w_ (diffusion resistance) are shown in Figure 2d. It is observed that 𝑅_ohm_ of four batteries changes minimally, indicating that the electrolyte resistance and contact resistance between electrode and current collector are not significantly affected, while 𝑅_sei_ increases with the degree of overcharge, meaning that overcharge leads to SEI thickening and increased deposition of side reaction products, and resulting in aggravated interface passivation. In contrast, 𝑅_sei​_ of over‐discharge batteries decreases, possibly due to the cracking or partial dissolution of SEI layer at low potentials, leading to reduced impedance. Moreover, 𝑅_ct_ increases slightly under mild over‐discharge, which is related to excessive extraction of sodium ions from anode material, hindering charge transfer due to reduced electrode surface reaction rate. Conversely, 𝑅_ct_ decreases with increased overcharge, potentially due to improved conductivity of side reaction products at the interface and localized activation of electrode. However, this represents only the impact of single electrical abuse on charge transfer, changes in 𝑅_ct_ under prolonged overcharge require long‐term abuse test. 𝑅_w_​ shows no significant differences among the four batteries, indicates that single mild electrical abuse does not markedly affect sodium‐ion diffusion behavior in electrode materials.

Figure 2e presents the relaxation voltage of four batteries within 2 h after different electrical abuse events. Both levels of overcharge result in elevated relaxation voltages, while over‐discharge leads to a decrease. More specifically, the relaxation voltage increases significantly by ≈0.2 V between fresh and 110% SOC overcharge batteries, whereas the increase from 110% SOC to 120% SOC overcharge is only ≈0.05 V. During cycling, especially under overcharge condition, some sodium ions fail to fully insert into the anode due to insufficient anode pore sites or rapid migration, leading to sodium plating on anode surface. Sodium plating reacts with the electrolyte, and slight voltage drop during relaxation process is caused by continuous side reactions involving the plated sodium. To further clarify the relationship between electrical abuse and sodium plating, DV curves of relaxation voltage are presented in Figure 2f. The local minima on DV curve represent the end of reaction between plated sodium and electrolyte. The DV peaks can be used to analyze the relationship between different levels of electrical abuse and completion time of sodium plating reactions.^[^ ^38^, ^39^ ^]^ It can be observed that the more severe overcharge, the earlier DV peak appears, while DV peak after over‐discharge occurs later than that of the fresh battery. The occurrence of DV peak is generally positively correlated with the amount of reversible sodium, representing complete consumption of reversible plated sodium. This indicates that overcharge gradually exacerbates sodium plating on anode surface, while over‐discharge leads to the dissolution of plated sodium. Therefore, by analyzing relaxation voltage of abused batteries, the content of reversible sodium on anode surface can be quantified non‐destructively.

### 2.2 Effect of Electrical Abuse on Thermal Stability of SIBs

In summary, mild electrical abuse has certain impact on electrochemical performance of SIBs. Overcharge temporarily increases capacity but exacerbates interfacial passivation and raises impedance, while over‐discharge weakens interfacial stability. Moreover, polarization changes and accumulation of sodium plating on the anode surface induced by mild electrical abuse may pose potential risks. Therefore, it is necessary to further investigate the specific impact and evolution mechanisms of mild electrical abuse on the thermal stability of SIBs.

#### 2.2.1 Heat Generation of SIBs

**Figure 3:**
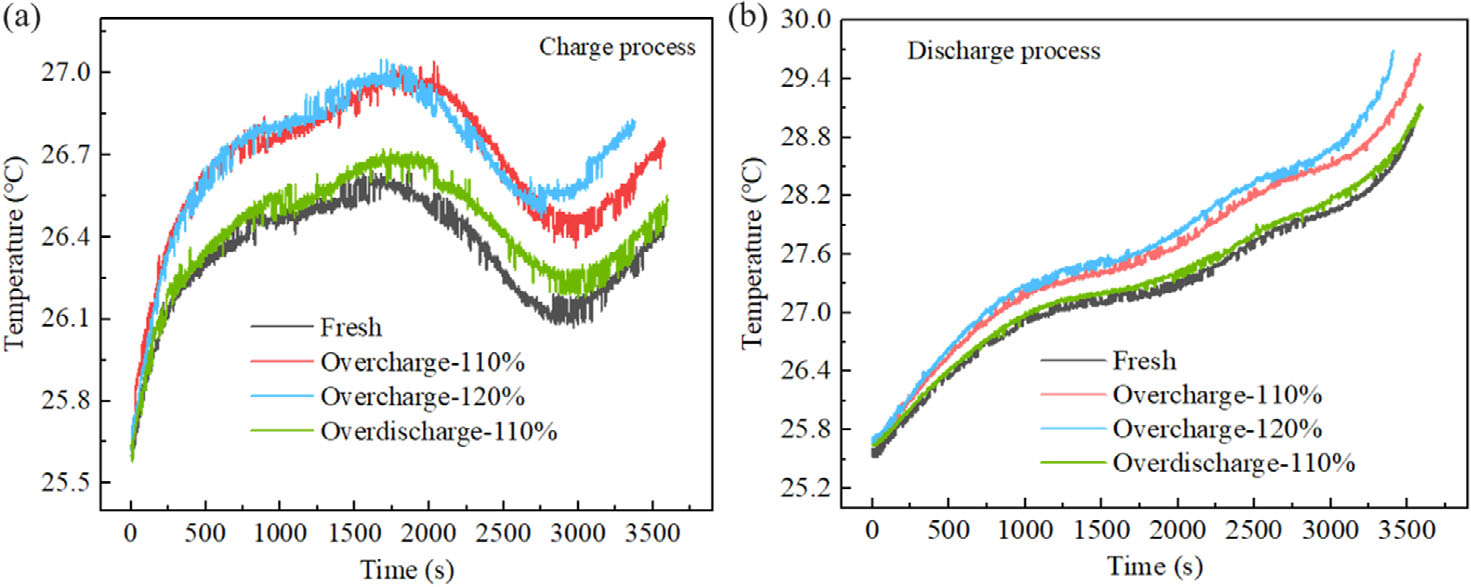
Comparison of charge and discharge temperature before and after electrical abuse. a) Temperature comparison during charge. b) Temperature comparison during discharge.

The battery generates heat during its normal cycle. By placing SIBs in the temperature‐controlled chamber to maintain constant ambient temperature, the rate of heat production inside the battery can be reflected by measuring the temperature on its surface. **Figure** **3** illustrates temperature rise differences during cycling for four batteries to evaluate the impact of mild electrical abuse on battery heat generation.

Figure 3a compares the temperature rise during charge for four batteries. It can be seen that the battery subjected to mild over‐discharge exhibits minimal temperature differences compared to the fresh battery, while overcharged batteries display more noticeable temperature rise differences, with a maximum temperature difference of 0.5 °C. Figure 3b presents similar trends during discharge, where the temperature rise curves of over‐discharged battery and the fresh battery nearly overlap. In contrast, the overcharged batteries show significant temperature rise differences, especially the battery subjected to 120% SOC overcharge, which exhibits a temperature rise ≈0.8 °C higher than the fresh battery. Therefore, it can be concluded that a single mild over‐discharge does not significantly impact the battery's subsequent cycle temperature, while an overcharge will significantly increase the cycle temperature of the battery.

#### 2.2.2 Thermal Runaway Behavior of SIB After Electrical Abuse

Batteries subjected to mild electrical abuse exhibit significant differences in cycling temperatures, indicating that abuse behaviors alter their thermal stability. If overcharge occurs without proper control, it can directly trigger severe TR. Therefore, it is crucial to understand the trend of thermal evolution of batteries after experiencing electrical abuse.

Figure S1 (Supporting Information) illustrates the typical voltage, temperature, and stress evolution of an NFM SIBs during overcharge‐induced TR. During overcharge, the battery first experiences internal gas generation, leading to swelling. This is followed by a rapid expansion phase, during which the battery reaches its physical limit and vents through small rupture, causing the 1st gas release, accompanied by noticeable increases in voltage and temperature. When the energy inside the battery accumulates to a certain extent, the 2nd exhaust occurs, and TR is triggered along with the sudden rise in temperature and rapid drop of voltage. Detailed stages and internal mechanisms of TR from LIBs can be found in our previous study.^[^ ^15^ ^]^

**Figure 4:**
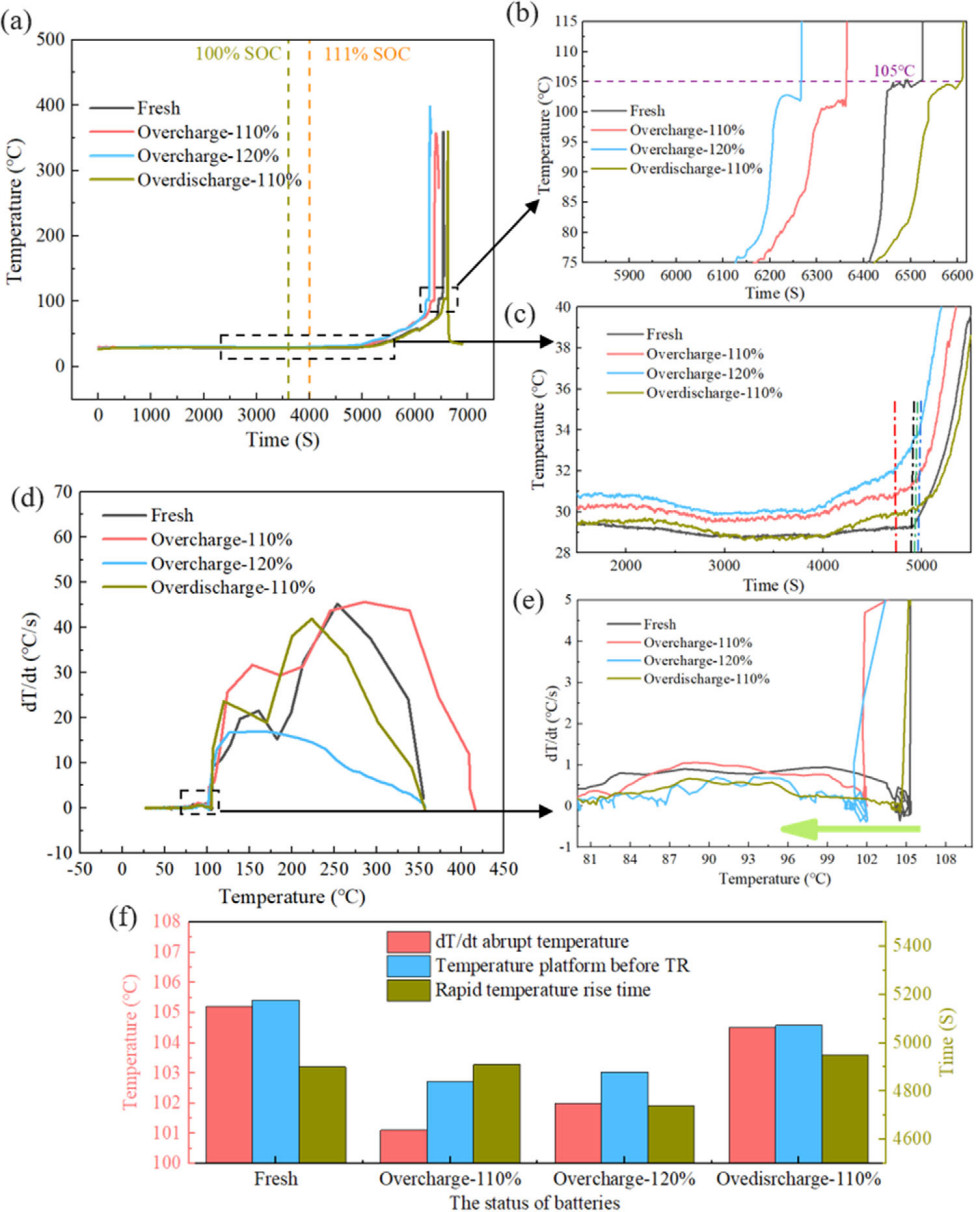
Thermal characteristics evolution of overcharge induced thermal runaway of battery after electrical abuse. a) Temperature evolution trend of thermal runaway process after electrical abuse. b) Differences in temperature platforms. c) The difference of inflection points of temperature change. d) Temperature rise rate comparison. e) Comparison of thermal runaway trigger temperatures. f) Summary of three kinds of data related to temperature.

The thermal evolution trends under overcharge‐induced TR for batteries subjected to varying degrees of electrical abuse are shown in **Figure** **4**. Mild electrical abuse significantly affects battery heat generation. Figure 4a presents the temperature profiles during overcharge‐induced TR of four batteries. Both levels of overcharge cause earlier TR onset, while over‐discharge has minimal impact on overall temperature changes. A temperature plateau occurs before TR, primarily due to endothermic processes of separator and binder melting at high temperatures. When the generated and absorbed heat are balanced, a temperature plateau appears.^[^ ^40^ ^]^ Figure S2 (Supporting Information) shows DSC analysis of separator fusion and binder decomposition, with pronounced endothermic peaks ≈150–160 °C. The measured temperature platform is ≈100 °C because the thermocouple is arranged on the battery surface, so the measured temperature is lower than actual internal temperature. Figure 4b shows that the temperature plateau for overcharged batteries not only occurs earlier but also decreases by ≈2 °C. Additionally, Figure 4c indicates that the trigger time for the temperature inflection point is significantly earlier at 120% SOC overcharge. These results suggest that the thermal stability of battery materials decreases after overcharge, while over‐discharge batteries are almost unaffected.

Figure 4d illustrates the temperature rise rate throughout TR. All batteries meet the TR trigger threshold ≈100 °C. Figure 4e is the zoomed‐in view and reveals that fresh and over‐discharged batteries trigger TR ≈105 °C, while overcharged batteries exhibit earlier onset, consistent with the temperature plateau trend. Figure 4f summarizes thermal parameters affected by electrical abuse. Mild overcharge not only lowers TR onset temperature but also advances and lowers temperature plateau. Furthermore, temperature inflection point during rapid heating occurs earlier with higher overcharge levels, particularly at 120% SOC. Conversely, mild over‐discharge has minimal impact on these signals.

### 2.3 Postmortem Analysis

The previous non‐destructive in situ tests and TR experiments have clarified macroscopic impacts of electrical abuse on SIBs. The degradation of material not only directly affects electrochemical performance of the battery, but may also further weaken its thermal stability and overall safety. Therefore, conducting an in‐depth investigation into the evolution mechanisms of battery materials under electrical abuse conditions is essential for uncovering the intrinsic relationship between performance degradation and changes in safety characteristics.

#### 2.3.1 Characterization of Cathode Electrode Materials

During the charging process, the layered cathode material will lose oxygen and sodium atoms, causing the c‐axis of the crystal structure to become smaller, but as long as it is within the allowable sodium de‐intercalation range, it will not cause structural changes. Once an overcharge occurs, the cut‐off voltage will increase, causing more sodium to be de‐intercalated from the cathode, resulting in an increase in the internal energy of the system. The stress changes in the crystal structure at the atomic level will induce the formation of micro cracks. The most direct impact of this crack is that it causes battery capacity attenuation and cycle performance degradation. In severe cases, it will even affect the thermal stability of the material.

**Figure 5:**
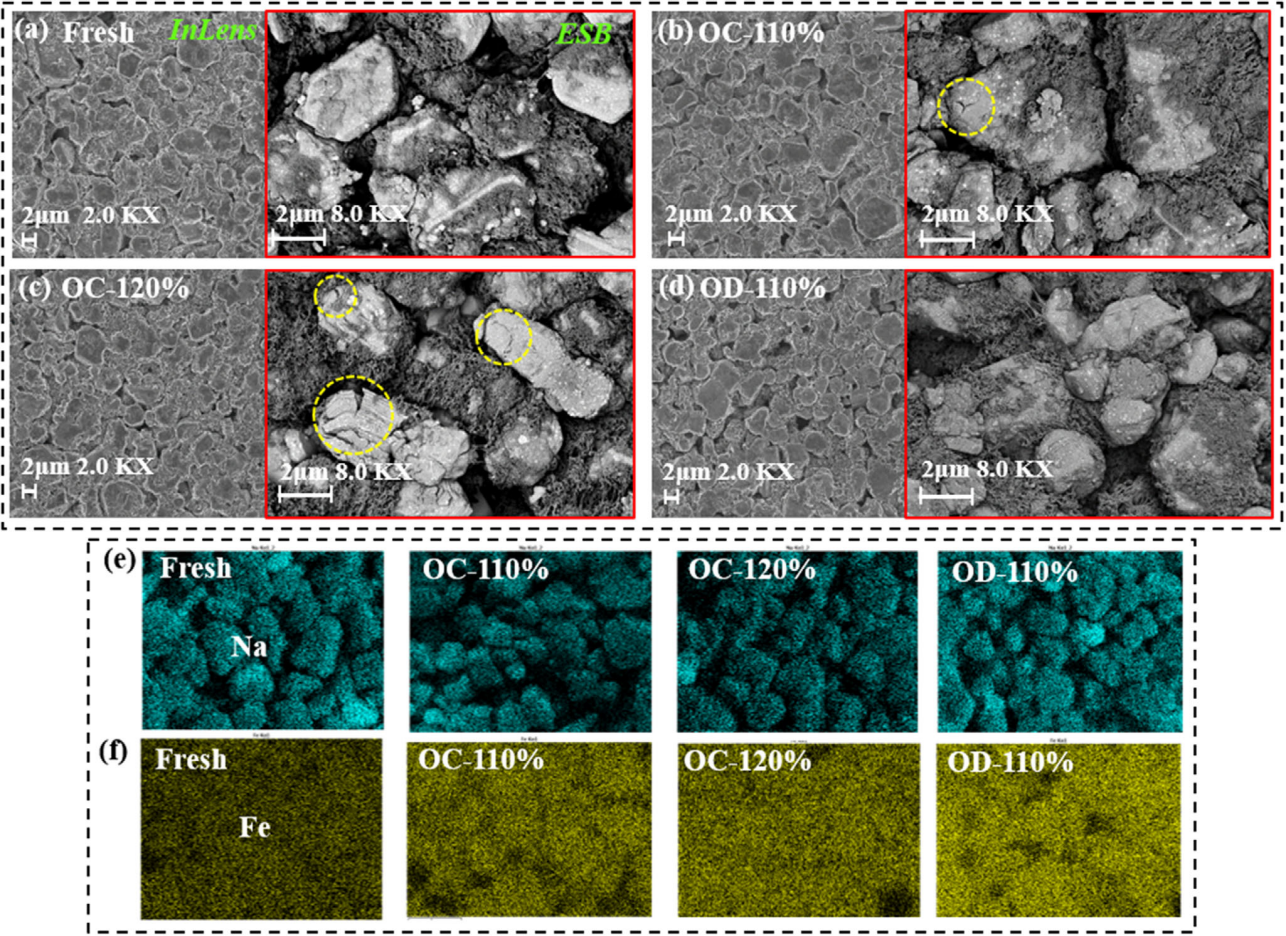
SEM and EDS‐mapping test results of cathode materials with different electrical abuse. a) Surface morphology of fresh battery under Inlens and ESB detectors. b) Overcharge 110% SOC battery. c) Overcharge 120% SOC battery. d) Over‐discharge 110% SOC battery. e) Na content distribution on an electrode surface. f) Fe content distribution on the electrode surface.

To investigate the degradation mechanisms of battery materials induced by electrical abuse, four cells subjected to mild electrical abuse and then charged to a fully charged state are disassembled in an argon‐filled glovebox. Advanced characterization techniques are used to analyze morphology, elemental composition and distribution, crystal structure, and thermal properties of the electrodes. **Figure** **5**a–d shows the surface morphology of electrodes from fresh batteries, overcharge batteries at 110% and 120% SOC, and −110% SOC over‐discharge batteries, respectively. Each image set includes 2.0kX magnification Inlens images and 8.0kX magnification ESB images. The backscattered electron detector provides high‐contrast volumetric imaging, enabling the identification of details that may be challenging to observe in Inlens images. From these images, the fresh electrode material exhibits an intact overall structure, with smooth, crack‐free particle surfaces. For the four electrodes subjected to electrical abuse, the overall structures remain intact without noticeable collapse. However, ESB images reveal that the electrode material surface of the 110% SOC overcharged battery shows minor cracks, while significant cracking and partial particle fracture are observed for the 120% SOC overcharged battery. In contrast, the surface of the over‐discharged electrode exhibits almost no visible cracks. This indicates that, for NFM cathode materials, overcharge causes greater damage to electrodes than over‐discharge, even under the same level of electrical abuse.

Figure 5e,f display Na and Fe elemental distribution results for four cathode materials under different electrical abuse conditions, with detailed Inductively Coupled Plasma (ICP) quantitative data provided in Table S1 (Supporting Information). From Na elemental distribution, it is observed that at 110% SOC overcharge, elemental content on cathode surface shows minimal changes. However, at 120% SOC overcharge, Na element content decreases significantly. In contrast, the over‐discharge battery exhibits a slight increase in Na content. This is because overcharge leads to excessive de‐intercalation of Na from cathode, while over‐discharge causes excessive intercalation of Na into cathode. Severe de‐intercalation or intercalation behaviors result in irreversible loss of active sodium. Additionally, Fe element content increases in all electrodes after electrical abuse, with the increase being particularly pronounced for over‐discharged electrodes. This suggests that metal dissolution is the critical factor contributing to battery capacity decline.

**Figure 6:**
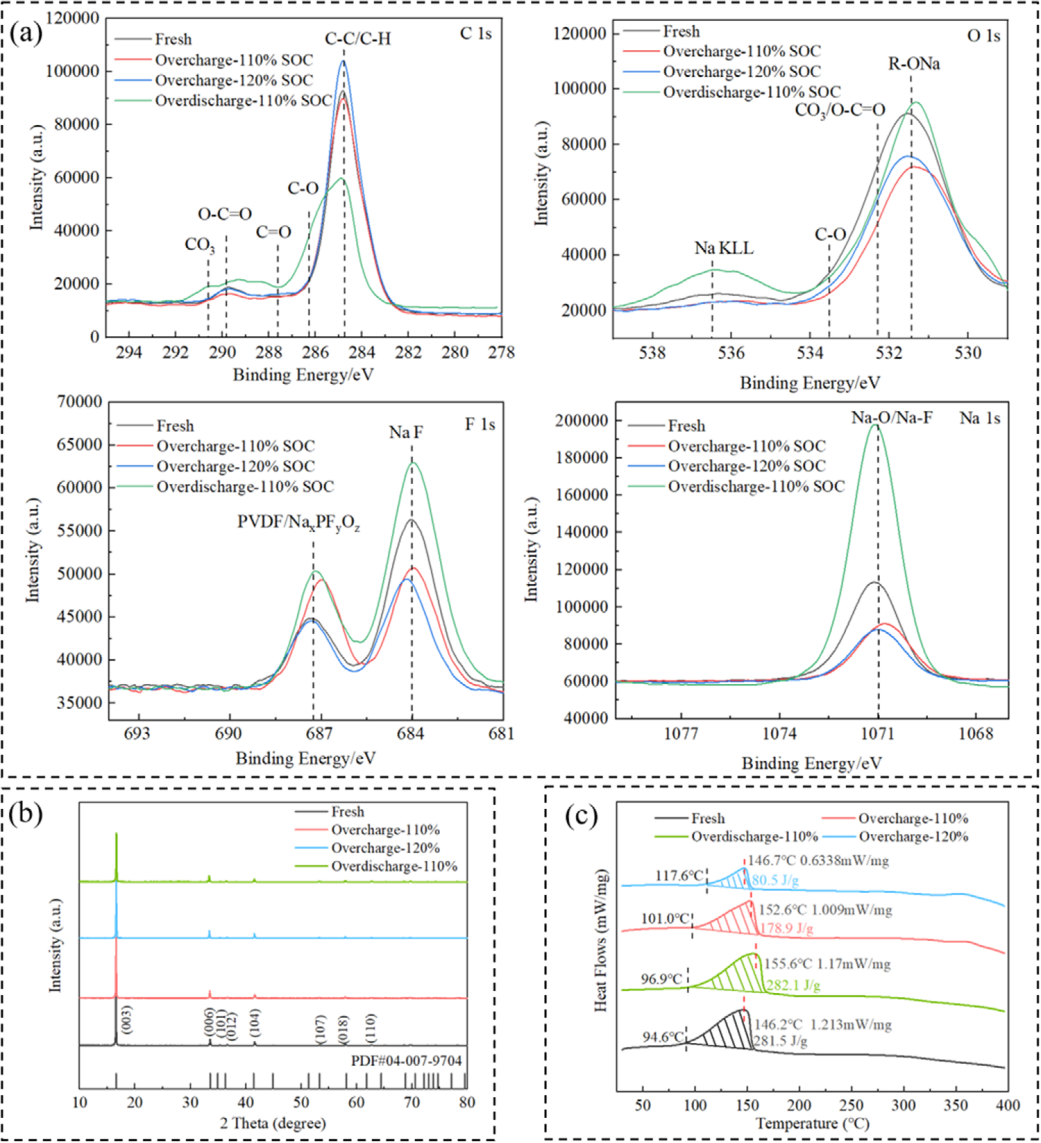
XPS, XRD, and DSC test results of cathode materials with different electrical abuse. a) XPS spectra of C, O, F, and Na elements. b) XRD test results. c) DSC test results.

**Figure** **6**a shows XPS results for C 1s, O 1s, F 1s, and Na 1s spectra of four electrode materials. In C 1s spectrum, the peak at 284.8 eV corresponds to C─C/C─H bonds in cathode electrolyte interphase (CEI) layer formed by the decomposition of conductive agent and electrolyte, while the peak at 290.3 eV comes from CO_3_, which originates from compounds such as Na_2_CO_3_. The four peaks between 286 and 291 eV indicate that surface oxidation reactions occur after electrical abuse, resulting in the formation of oxygen‐containing compounds. In O 1s spectrum, the peak ≈530.3 eV corresponds to lattice oxygen, the peak at 532.3 eV originates from carbonate species corresponding to CO_3_/O─C≐O groups, and the peak at ≈536.5 eV is attributed to Auger peak of Na. During overcharge, intensities of these peaks decrease, possibly due to decomposition and consumption of reactive species on the cathode surface, leading to the formation of compounds not primarily containing oxygen, such as gases. In contrast, during over‐discharge, intensities of these peaks increase, suggesting the generation of large amounts of oxygen‐containing compounds. In F 1s spectrum, the peak at 684 eV corresponds to NaF in CEI layer, and the peak at 687.3 eV arises from decomposition products of binder PVDF and sodium salts. The NaF peak decreases during overcharge, which may also be related to the decomposition of electrolytes and generation of volatile gases. During over‐discharge, a significant amount of fluoride‐containing species is formed, possibly due to reduction reactions induced by over‐discharge. The Na 1s spectrum shows a peak at 1071 eV, indicating that overcharge leads to a decrease in sodium content in cathode material, while over‐discharge significantly increases it.

Figure 6b shows XRD results, indicating that after electrical abuse, the XRD pattern of the cathode material does not exhibit significant changes compared to the fresh battery. The main diffraction peaks corresponding to the (003), (006), (101), (012), (104), and (110) crystal planes remain clearly visible, suggesting that the crystal structure of cathode material is generally well‐preserved. Although cathode material maintains relatively high structural stability under mild electrical abuse, differences in lattice parameters can still be observed from the results in **Table** **1**. During overcharge, the a‐axis and b‐axis expand, while the *c*‐axis shrinks, likely due to the expansion of material in plane direction and contraction in vertical direction during overcharge. This is probably caused by crystal distortion due to ion transition intercalation. In contrast, during over‐discharge, the a‐axis and b‐axis shrink, and the *c*‐axis expand, indicating lattice contraction in the material during over‐discharge. Moreover, the effect of overcharge on lattice parameters is more significant than that of over‐discharge under the same level of electrical abuse.

**Table 1:** Lattice parameters of cathode materials.

	*a* [Å]	*b* [Å]	*c* [Å]	*c*/*a*
Fresh	2.8884	2.8884	16.6353	5.7593
Overcharge‐110%	2.8895	2.8895	16.6306	5.7555
Overcharge‐120%	2.9004	2.9004	16.6268	5.7325
Overdischarge‐110%	2.8885	2.8885	16.6879	5.7773

Figure 6c presents DSC results, showing the impact of electrical abuses on heat release behavior of cathode material. It is observed that the fresh electrode exhibits distinct exothermic peak ≈146.2 °C, corresponding to the maximum heat flow intensity of 1.213 mW mg^−1^. Overcharge and over‐discharge at ‐110% SOC do not produce significant effects on the heat release of electrode material. However, when overcharged to 120% SOC, heat release of cathode material even decreases, and the initial reaction temperature increases. These results indicate that under mild electrical abuse, heat release from cathode material does not make a significant contribution to temperature change during the overcharge‐induced TR process.

#### 2.3.2 Characterization of Anode Electrode Materials

**Figure 7:**
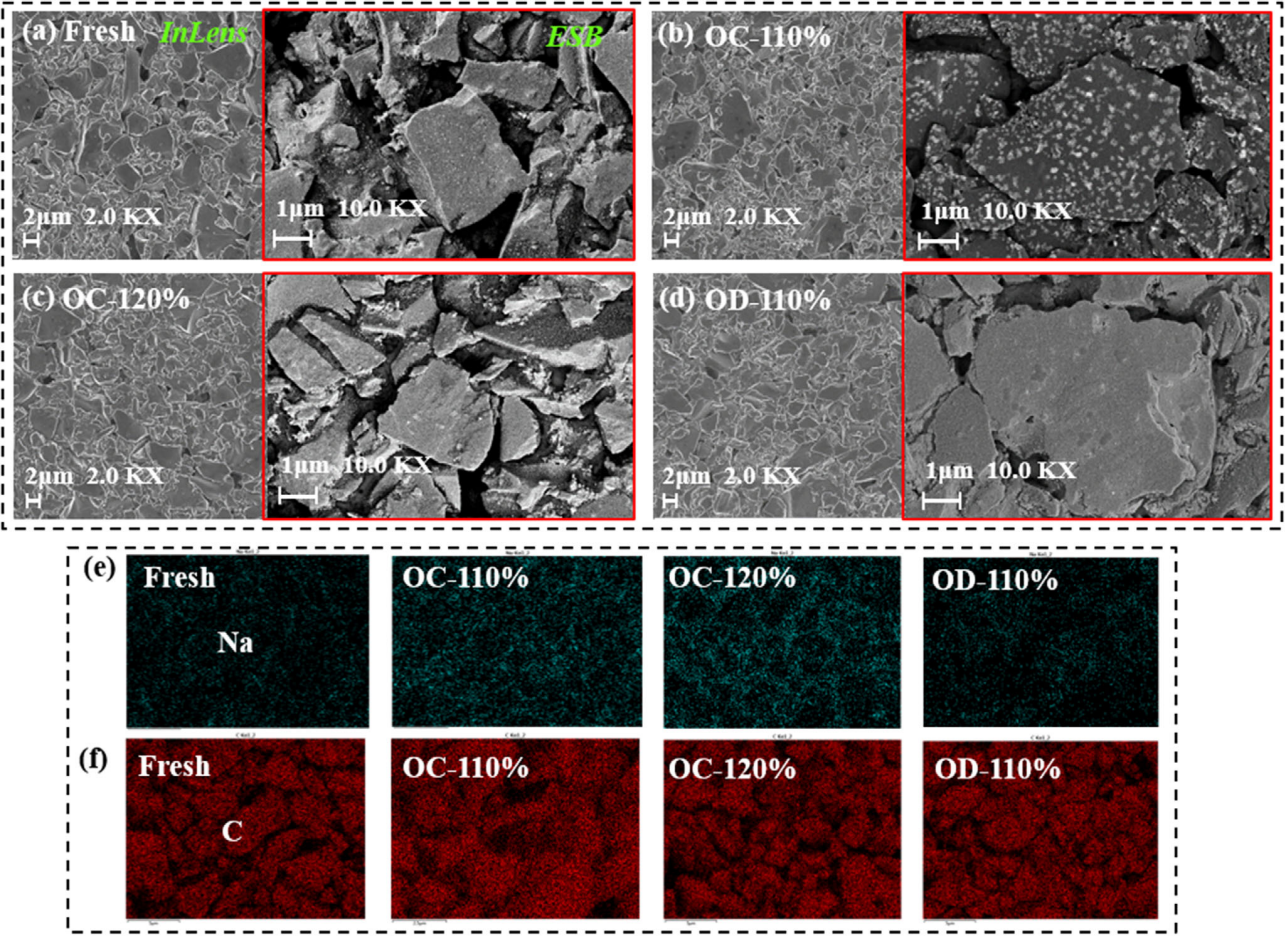
SEM and EDS‐mapping test results of anode materials with different electrical abuse. a) Surface morphology of fresh battery under Inlens and ESB detectors. b) Overcharge 110% SOC battery. c) Overcharge 120% SOC battery. d) Over‐discharge 110% SOC battery. e) Na content distribution on an electrode surface. f) C content distribution on electrode surface.

**Figure** **7**a–d shows the surface morphology of four anode materials under different electrical abuse conditions. Compared to fresh materials, no significant structural changes are observed in electrode materials after mild electrical abuse in the Inlens images. The particle surfaces remain intact, with no signs of breakage or pulverization. However, the ESB images reveal notable differences in sodium plating levels on the surfaces of four electrodes. In Figure 7a, the fresh material surface is relatively smooth but a few white spots are visible, which through elemental analysis, are confirmed to be sodium metal, indicating that small amount of ion enrichment occurs during normal cycle. In Figure 7b, overcharge at 110% SOC, obvious white deposits appear on the anode surface. In Figure 7c, anode surface becomes fully covered with sodium deposits at 120% SOC, indicating significant sodium plating. In Figure 7d, during over‐discharge, anode surface shows almost no visible evidence of sodium deposition, suggesting that over‐discharge depletes the residual sodium plating on anode surface. Therefore, electrical abuse notably affects sodium plating on anode surface.

Figure 7e,f presents the distribution results of Na and C elements in four anode materials under different electrical abuse conditions. The trend of Na element variation aligns with observations from SEM, overcharge increases the sodium content on the electrode surface, while over‐discharge reduces the Na content. The distribution trend of C elements has not changed significantly. Detailed anode ICP quantitative results are presented in Table S2 (Supporting Information).

**Figure 8:**
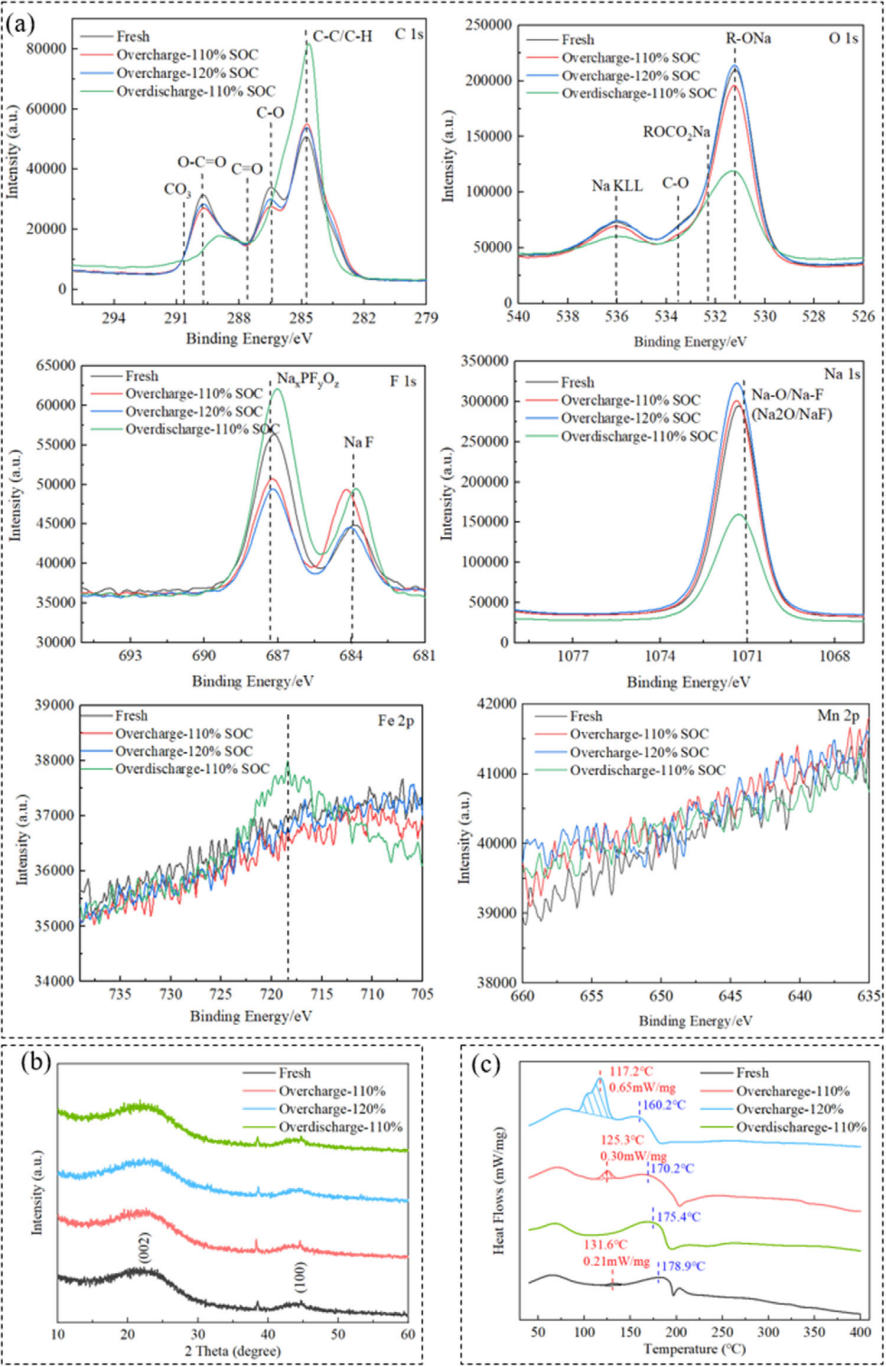
XPS, XRD, and DSC test results of anode materials with different electrical abuse a) XPS spectra of C, O, F, Na, Fe, and Mn elements. b) XRD test results. c) DSC test results.

**Figure** **8**a presents XPS results for C 1s, O 1s, F 1s, Fe 2p, Na 1s, and Mn 2p spectra of four anode materials. In C 1s spectrum, C─C/C─H bond peak is the primary peak, indicating that anode material is carbon‐based, with small amount of O─C═O, CO_3_, and other functional groups. After overcharge, an increase in the main peak and decrease in other functional groups suggest that organic by‐products generated from electrolyte decomposition might cover anode surface, SEI film thickens due to the formation of by‐products but is usually rough and structurally uneven, which increases the interface impedance. However, after over‐discharge, enhanced C─C/C─H signal is likely due to the exposure of hard carbon (HC) framework, as sodium ions transition de‐intercalation. The weakening of peaks related to organic components in the C 1s spectrum is mainly due to the reduction or dissolution of organic components in the SEI film at low potential. For O 1s spectrum, the peak at 531.2 eV corresponding to R‐ONa indicates that anode surface contains certain amount of organic salts, which are also significantly affected by electrical abuse. In F 1s spectrum, the NaF peak increases significantly after electrical abuse, indicating that electrolyte decomposition caused by electrical abuse produced more NaF. The Na 1s spectrum shows increase in Na‐O/Na‐F signal during overcharge and decrease during over‐discharge, further suggesting that overcharge and over‐discharge significantly impact sodium compound content on anode surface. In Fe 2p and Mn 2p spectra, the overall signal change is minimal, but noticeable Fe signal appears at −110% SOC during over‐discharge. This suggests that over‐discharge causes Fe ions dissolve out from cathode material and migrate to anode surface. The dissolve out of Fe can affect electrochemical interface reactions, lead to degradation in the battery's electrochemical performance and capacity.

Figure 8b presents XRD results for four anode materials, showing that there is little difference in the peak shapes between anodes of the battery after mild electrical abuse and the fresh battery. This indicates that anode structure remains intact and has not been damaged. Figure 8c shows DSC results, illustrating the impact of different electrical abuse conditions on the thermal release behavior of anode material. For the fresh anode, there is an exothermic peak at 178.9 °C. The over‐discharge anode shows minimal changes in the position and intensity of this exothermic peak. However, for the overcharge anode, the exothermic peak shifts to lower temperature, showing a clear trend of temperature advancement. This indicates that overcharge decreases the material's thermal stability, and the advancement of exothermic peak temperature can affect the timing of various trigger points during TR. It is noteworthy that the fresh anode exhibits 0.21 mW mg^−1^ exothermic peak in 100—150 °C range. After over‐discharge at 110% SOC, this peak disappears. After overcharge at 110% SOC, the exothermic peak reappears and its intensity increases to 0.3 mW mg^−1^. When overcharge reaches 120% SOC, the exothermic peak further increases, with the maximum heat flow intensity reaching 0.65 mW mg^−1^. This trend is strongly correlated with the degree of sodium plating observed in Figure 7. Moreover, more severe sodium plating also leads to the advancement of exothermic reaction temperature. Therefore, it is believed that the changes in intensity and position of exothermic peak caused by mild electrical abuse are related to sodium plating on the anode surface. Furthermore, during actual battery overcharge, cathode material, and electrolyte decompose at high temperatures and voltages to generate oxygen‐containing substances, and their contact with sodium will further aggravate this exothermic phenomenon, and the deposited sodium may reduce the activation energy of the electrolyte decomposition, thus accelerating the exothermic reaction.^[^ ^41^ ^]^

### 2.4 The Effects of Long‐Term Electrical Abuse Cycles on SIBs

The impact of single mild electrical abuse event on battery performance and safety characteristics is minimal. However, in practical applications, batteries typically undergo repeated or prolonged instances of electrical abuse, rather than single occurrence. Therefore, it is essential to conduct long‐term overcharge and over‐discharge testing to clearly define the degradation path of battery's capacity and safety characteristics under prolonged mild electrical abuse conditions. The appearance of the battery after 150 cycles of overcharge and over‐discharge is shown in Figure S3 (Supporting Information), and there is no obvious physical change compared with the fresh battery.

#### 2.4.1 Degradation Analysis of Electrochemical Performance

**Figure 9:**
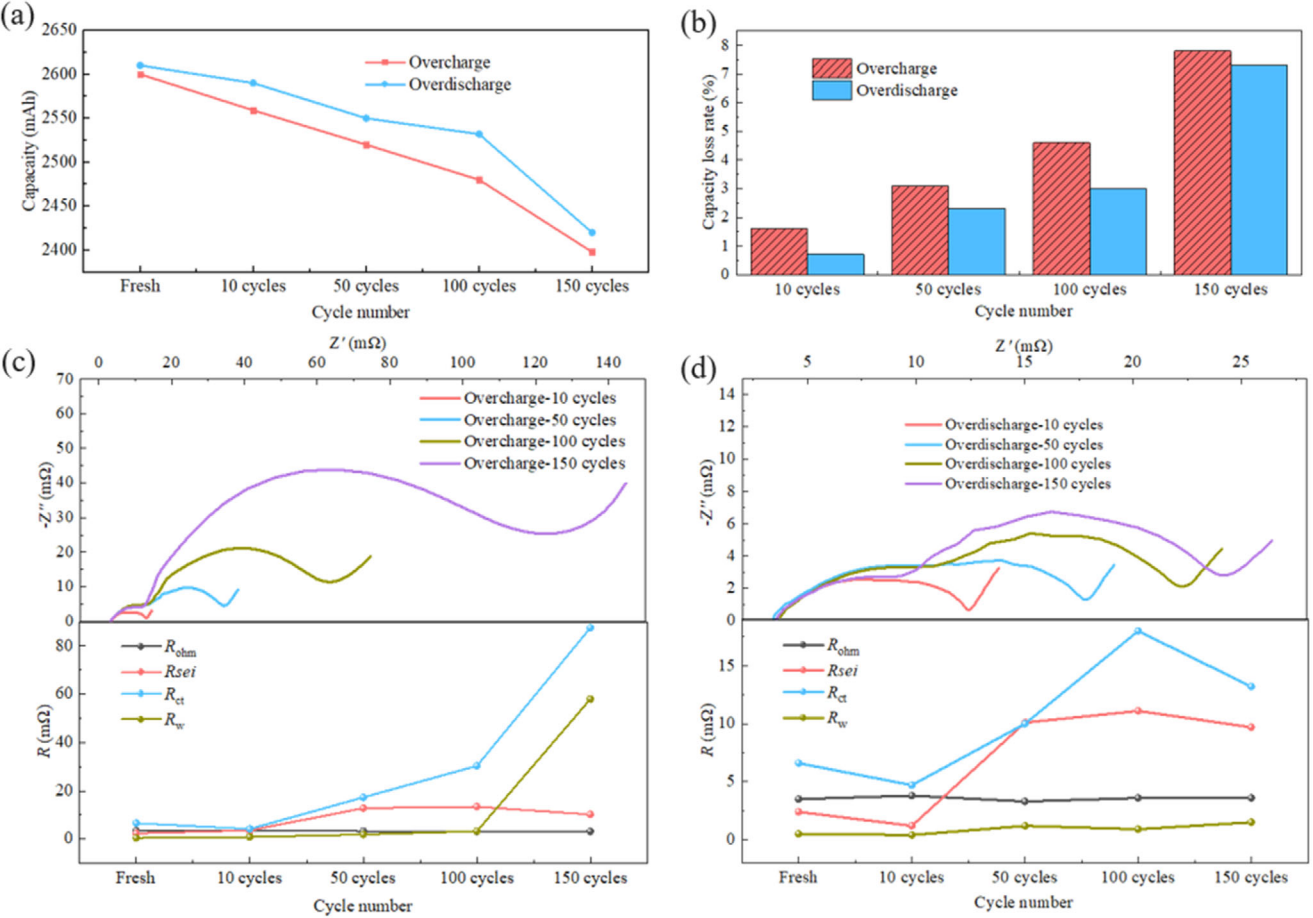
Capacity and EIS test results after different cycles of electrical abuse. a) Remaining capacity after electrical abuse. b) Capacity loss rate after electrical abuse. c) EIS curve and impedance fitting results of overcharge battery; (d) EIS curve and impedance fitting results of over‐discharge battery.

Through the previous study on single electrical abuse event of SIBs, it has been clearly shown that performance‐degraded batteries exhibit significant differences in their EIS curves and fitted impedance values compared to fresh batteries. To investigate the performance degradation mechanism of batteries after long‐term overcharge and over‐discharge, **Figure** **9** presents the capacity loss rate, EIS curves, and corresponding impedance values of fresh batteries and those subjected to 10, 50, 100, and 150 cycles of mild electrical abuse. As shown in Figure 9a, even after ten cycles of mild overcharge or over‐discharge at 110% SOC, the battery's capacity begins to show signs of degradation. As shown in Figure 9b, the capacity degradation rate increases with increased cycles. When the electrical abuse cycles reach 100, the capacity degradation caused by overcharge exceeds 5%, and after 150 cycles, the capacity loss caused by over‐discharge reaches ≈8%. In conclusion, the capacity degradation shows linear increase, with the overcharge‐induced capacity loss being higher than that induced by over‐discharge.

Figure 9c,d shows the changes in EIS curves and impedance values of the battery subjected to different cycle counts of mild overcharge and over‐discharge. It is evident that as the overcharge cycle count increases, the semi‐circle in EIS curve becomes significantly larger. Small changes in *R*_ohm_ during the cycles suggest that overall conductivity of the electrolyte, electrode, and current collector is not significantly affected. For *R*_sei_ during overcharge, the increase in the early stages of cycling indicates that SEI film gradually thickens under prolonged overcharge. However, *R*_sei_ stabilizes after 50 cycles. In contrast, during over‐discharge cycles, *R*_sei_ slightly decreases in early stages, which may be attributed to damage to SEI film caused by over‐discharge. Nevertheless, after 50 cycles, *R*_sei_ increases and stabilizes, suggesting that SEI film reorganizes and thickens under long‐term over‐discharge conditions. *R*_ct_ exhibits a downward trend in early cycles, similar to the results observed after single electrical abuse, but significantly increases after 50 cycles, indicating that electrochemical interface reaction activity is significantly reduced. This is consistent with SEM results of cathode in Figure 5, which suggest that the structure of active material deteriorates over time. Additionally, the increased sodium plating observed in anode SEM images in Figure 7 likely contributes to the rise in interfacial impedance. For *R*_w_, there is little change in the early stages of overcharge cycles, but it increases noticeably after 100 cycles, indicating that the diffusion of sodium ions is hindered at this point. This is likely related to the internal particle fracture, phase transitions, and damage to the crystal structure of electrode material. On the other hand, *R*_w_ during over‐discharge shows no significant change, suggesting that over‐discharge has less effect on the internal structure of electrode material. In summary, the impedance changes caused by overcharge are much more pronounced than those caused by over‐discharge. This indicates that prolonged overcharge significantly affects electrochemical performance and structural integrity of the battery, whereas over‐discharge has relatively smaller impact.

We believe that the linear capacity fade and nonlinear impedance evolution reflect the multi‐scale nature of battery degradation. Linear capacity fade is primarily due to the gradual loss of active sodium ions caused by irreversible side reactions. This loss accumulates over cycles, leading to a linear trend that correlates with cycle count. In contrast, the nonlinear increase in impedance reflects dynamic interfacial processes at both electrodes. Therefore, the linear capacity fade is time‐dependent, while the nonlinear increase in impedance is driven by interfacial evolution (SEI/CEI dynamics) and localized failure events (such as micro‐short circuits triggered by sodium dendrites), which exhibit threshold‐dependent nonlinearity.

The composition of the electrolyte also influences the EIS test results of the battery under long‐term electrical abuse cycling. In this study, the electrolyte used is 1 m NaPF₆ in EC: PC: DEC: EMC = 1:3:1:4 Vol% with 1% FEC. NaPF₆ is widely used in SIBs due to its moderate ionic conductivity. However, its slight hydrolysis under abuse conditions may produce acidic species such as HF, which can corrode electrode materials and form new by‐products, leading to interface instability and increased interfacial impedance. This is consistent with the increase in interfacial resistance *R*_sei_ observed after long‐term electrical abuse cycling. The addition of EC, due to its high dielectric constant and reductive decomposition tendency, promotes the formation of a stable SEI layer on the anode. While the addition of PC improves low‐temperature performance, its intercalation into the anode under abuse conditions may cause partial delamination and SEI instability, which could be one reason for the gradual increase in *R*_ct_ after long‐term electrical abuse cycling. Although the addition of DEC and EMC enhances the wettability of the electrolyte, reduces viscosity, and facilitates uniform current distribution, they may generate resistive substances such as polycarbonate under abuse conditions, which could explain the slight increase in low‐frequency *R*_w_ impedance.

#### 2.4.2 Thermal Stability Degradation Analysis

**Figure 10:**
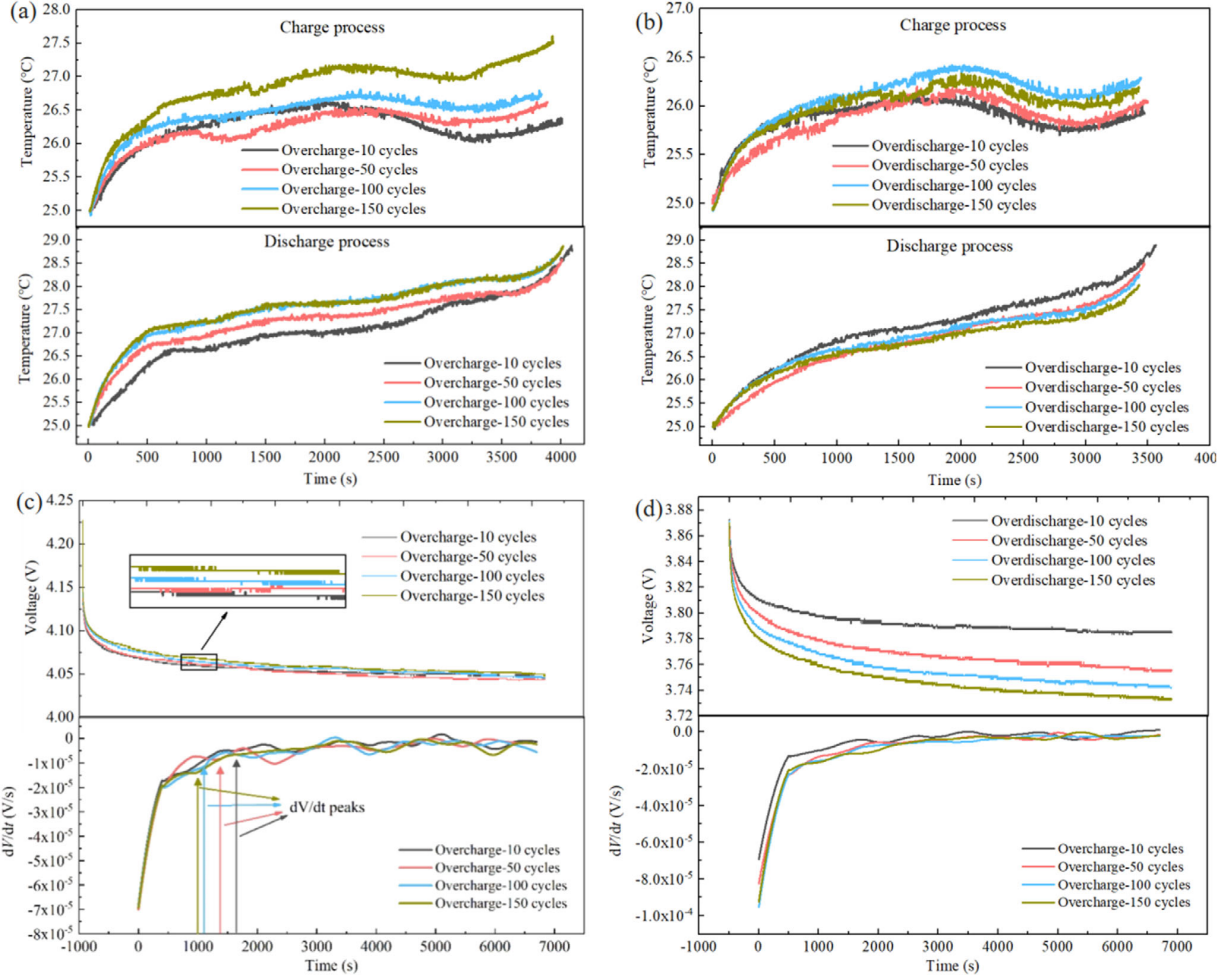
Comparison of battery charge and discharge temperature after different cycles of electrical abuse. a) Charge and discharge temperature of overcharge battery. b) Charge and discharge temperature of the over‐discharge battery. c) Comparison of relaxation voltage and DV curve of overcharge battery. d) Comparison of relaxation voltage and DV curve of the over‐discharge battery.

**Figure** **10**a,b compare cycling temperature variations of the battery subjected to two types of mild electrical abuse over different cycle counts. From Figure 10a, which shows the cycling temperature after overcharge, the temperature rise during charge after 10, 50, and 100 cycles of overcharge abuse remains relatively small. However, after 150 cycles, there is a significant increase in temperature. The discharge temperature rise shows a gradual increase after 10, 50, and 100 cycles, but after 150 cycles, the temperature rise becomes nearly constant. The maximum temperature difference exceeds 1 °C in all cases. Figure 10b presents the cycling temperature after over‐discharge. The temperature differences during both charge and discharge are smaller than those observed after overcharge, with the maximum temperature difference not exceeding 0.5 °C. Moreover, as the number of cycles increases, the temperature rise during discharge even shows a downward trend.

Previous research on single electrical abuse has shown that sodium plating on anode directly impacts temperature rise during cycling. Therefore, relaxation voltage (DV curve) is used to theoretically calculate the amount of sodium plating and establish a relationship between sodium plating and temperature rise. Figure 10c shows relaxation voltage and DV curves after overcharge for different cycle counts. From the zoomed‐in view of relaxation voltage, it can be observed that as the number of overcharge cycles increases, relaxation voltage curve gradually increases. Simultaneously, DV peak shifts progressively to the left with increased cycle count. Based on the previous explanation, this indicates an increase in sodium plating. Therefore, the growing amount of sodium plating will impact the temperature rise during overcharge cycles. Figure 10d presents relaxation voltage and DV curves after over‐discharge for different cycle counts. As the number of over‐discharge cycles increases, relaxation voltage gradually decreases. However, no distinct peaks are observed in the DV plot, making it difficult to identify sodium plating‐related information from over‐discharge DV curves. This suggests that long‐term over‐discharge cycles cause sodium plating on anode surface to dissolve. Therefore, for SIBs, even if they are subjected to long‐term over‐discharge cycles, they will not have significant impact on the temperature rise of the cycle process.

## 3 Experimental Section

### 3.1 Details of Tested Sodium‐Ion Battery

In this study, pouch‐type SIBs with a nominal capacity of 2.6 Ah were used for the analysis, all test batteries were provided by Huzhou Horizontal Na Energy Technology Co., Ltd. Using new batteries for the experiments avoids confounding variables associated with aging (e.g., SEI accumulation, loss of active materials), thereby allowing a clearer assessment of the side effects induced solely by abuse conditions. The batteries utilized layered oxide NFM as cathode material and HC as anode material. Table S3 (Supporting Information) provides selected geometric and electrochemical parameters of the NFM SIBs. Before starting experiments, battery consistency testing was conducted. The batteries were placed in a thermostat (NEWARE MHW‐25‐S‐16CH, China) set to an ambient temperature of 25 °C. Using the Battery testing system (BTS, NEWARE BTS‐20V100A, China), each fresh battery undergoes ten precycling processes. The average discharge capacity over the ten cycles was taken as the actual capacity of the battery. The consistency testing steps are shown in Table S4 (Supporting Information).

### 3.2 Battery Grouping and Experimental Setup

Ten SIBs with good consistency were selected for the experiments, which were divided into three groups as listed in **Table** **2**. The table shows the actual capacities of the ten batteries after consistency testing, along with the corresponding test conditions and testing objectives for each battery. In the 1st group, four batteries are normally charged at constant‐current (CC) mode, at 1C‐rate (2.6 A) to 100% SOC (2.6 Ah), overcharge to 110% SOC (2.86 Ah), overcharge to 120% SOC (3.12 Ah), and over‐discharge to −110% SOC (−2.86 Ah). Overcharge was conducted from 1.5 V to 110% and 120% SOC of the rated capacity as the cutoff condition, while over‐discharge was conducted from 3.9 V to 110% SOC of the rated capacity as the cutoff condition. The corresponding overcharge and over‐discharge curves are shown in Figure S4 (Supporting Information).

**Table 2:** Battery grouping information and test purpose.

Group No.	Battery No.	Actual capacity [Ah]	Electrical abuse details	Test item	Test purpose
1	1	2.61	100% SOC, Overcharge to TR	Capacity, Temperature, IC, DV, EIS, TR	Capacity, temperature rise, and TR test after a single electrical abuse
	2	2.63	110% SOC, Overcharge to TR		
	3	2.59	120% SOC, Overcharge to TR		
	4	2.61	−110% SOC, Overcharge to TR		
2	5	2.60	100% SOC	SEM‐EDS, XRD, XPS, DSC	Material characterization after a single electrical abuse
	6	2.57	110% SOC		
	7	2.59	120% SOC		
	8	2.62	−110% SOC		
3	9	2.61	110% SOC (10/50/100/150 cycles)	Capacity, EIS, Temperature, DV	Effects of long‐term cycle electrical abuse on capacity and temperature rise
	10	2.62	−110% SOC (10/50/100/150 cycles)		

These batteries then undergo capacity testing, cycle temperature testing, IC and DV testing. The IC test was used to identify the phase transition and side reaction inside the battery by analyzing the differential of the charge and discharge curve, and the DV test was used to detect the phase transition and aging mechanism of the electrode material by analyzing the voltage change rate with capacity. Finally, these four batteries were subjected to overcharge‐induced TR experiments to investigate trigger SOC and temperature variations at critical nodes during the overcharge to TR process after electrical abuse. The 2nd group undergoes three levels of electrical abuse similar to the first group, but these batteries were subsequently disassembled for morphology observation, lattice structure analysis, elemental analysis, and heat generation analysis. In the 3rd group, two batteries were subjected to overcharge and over‐discharge experiments at 110% SOC for 10, 50, 100, and 150 cycles. This was conducted to explore the degradation mechanisms and heat generation characteristics of batteries under prolonged electrical abuse. Figure S5 (Supporting Information) illustrates the principles of electrochemical and material characterization tests performed in this study.

### 3.3 Electrochemistry and Temperature Test

After precycling, the 1st group batteries were subject to electrochemical and temperature tests. Table S5 (Supporting Information) shows detailed protocols for electrochemical and temperature tests, including cut‐off voltage and SOC for electrical abuse, and rest time. For the over‐discharge batteries, standard charge process was performed immediately after over‐discharge to obtain voltage and capacity data during charge. Moreover, nondestructive IC analysis was employed to conduct electrochemical tests on the abused batteries. The IC curve was derived by calculating capacity change within a unit voltage interval, and the peak positions and magnitudes in the curve were analyzed to identify battery degradation mechanisms.^[^ ^42^, ^43^, ^44^ ^]^ Immediately after completing the charge process, 2‐h voltage relaxation of each battery was recorded. Furthermore, an improved DV analysis was utilized to investigate sodium plating during the electrical abuse process,^[^ ^38^, ^45^ ^]^ since no current flows during the relaxation period, differential capacity data cannot be obtained directly. Instead, a derivative of relaxation voltage curve was calculated using a time vector, representing the slope of the voltage curve. The improved DV curve for relaxation voltage was calculated using Equation (1). To achieve higher precision in voltage and capacity data acquisition during IC and DV test, the sample interval was set to 0.1 s. To reduce noise, curves were subsequently smoothed during post processing.

(1) $$V\left(v,t\right)=\frac{dV}{dt}$$

EIS test was conducted on fresh batteries and electrically abused batteries using an electrochemical workstation (EnergyLab XM, USA), and EIS curves were then fitted with construct equivalent circuit models to analyze impedance changes. In obtaining EIS curves, the test frequency ranges from 10 kHz to 1 mHz with an amplitude is 10 mV. Subsequently, standard cycling was performed on all four batteries to evaluate capacity changes after an electrical abuse event. During the cycling process, surface temperature variations of the batteries within the thermostat were recorded using thermocouples attached to battery surfaces.

### 3.4 Thermal Runaway Experiment

The 2nd group of four SIBs undergoes overcharge‐induced TR experiments after experiencing the same electrical abuse conditions as the 1st group. As shown in Figure S5 (Supporting Information), three K‐type thermocouples were attached to the battery surface to measure temperature at different locations, and high‐sensitivity, low‐stiffness strain sensor (with a resolution and accuracy of 0.1 and 1.0 µm, respectively) was used to monitor surface expansion during the overcharge process. To ensure the safety of experimental equipment and personnel, the battery was placed in an explosion‐proof chamber (NEWARE JD‐6009B, China). In the test, the battery was connected to BTS and subjected to CC overcharge at 1C rate, where voltage, current, and other signals were recorded, while a high‐speed camera captured the entire TR process. During the TR experiments, no upper limit was set for overcharge, and the test was stopped until TR was triggered.

### 3.5 Long‐Term Cycle Electrical Abuse Test

The 3rd group of two batteries was subjected to long‐term cycling at 1C‐rate under 110% SOC overcharge and −110% SOC over‐discharge conditions, respectively. With every 50 cycles, the cycling was paused to perform a standard cycle, during which battery capacity, cycle temperature rise, EIS curves, and relaxation voltage DV curves were collected. After the measurements, the next phase of 50‐cycle abuse testing begins. The long‐term abuse testing includes 10, 50, 100, and 150 cycles, with all cycling performed inside the temperature‐controlled chamber to ensure operation consistency under 25 °C environment.

### 3.6 Battery Disassembly and Material Characterization

For this purpose, the fully charged fresh battery and three abused batteries are disassembled in an argon‐filled glovebox (H₂O and O₂ < 0.1 ppm) using ceramic‐insulated scissors to extract cathode, anode, and separator materials for characterization. The disassembled materials were then stored and transferred in argon‐filled glass containers. To prevent material alterations, all tests were performed within 1h of disassembly.

A field‐emission SEM (Genimi SEM 500, England) was used to observe morphological changes in electrode materials and generate high‐resolution images. EDS was employed to analyze elemental composition. XPS (Thermo NEXSA G2, England) was selected to determine the chemical composition of electrodes, with binding energy calibrated using C1s peak of contaminant carbon at 284.80 eV. XPS technology uses X‐rays to excite core electrons of surface atoms in the sample and analyzes the elemental composition and chemical states by measuring the binding energy of the emitted photoelectrons. XRD (Rigaku SmartLab SE, Japan) was utilized to examine the electrode structure, with a scanning step size set at 10°min^−1^. XRD technology uses the diffraction of X‐rays by the crystal lattice of materials to analyze the crystal structure and phase composition based on Bragg's equation. Thermal properties of electrodes, separators, and binders were analyzed using DSC (TA instrument Q2000, USA) with a heating rate of 10 K min^−1^. DSC testing measures the heat flow difference between the sample and the reference under programmed temperature control to analyze endothermic and exothermic processes. All tests were conducted in a non‐air‐exposed environment to ensure the accuracy and reliability of the results.

## 4 Discussion

This study investigates the impact of mild electrical abuse on the safety characteristics of SIBs, **Table** **3** provides a detailed overview of the degradation mechanisms and pathways of SIBs under mild overcharge and over‐discharge for two different modes of electrical abuse. Advanced characterization techniques reveal that overcharge significantly affects the electrochemical performance and thermal stability of SIBs, for example, overcharge can lead to SEI layer thickening, sodium plating on the anode, reduced thermal stability, and increased impedance and temperature rise during cycling. In contrast, over‐discharge causes sodium plating dissolution, with insignificant effects on thermal stability and structural integrity. Under long‐term electrical abuse cycles, overcharge further accelerates the degradation process, while over‐discharge results in slower degradation behavior. In summary, mild overcharge poses a greater threat to the performance and safety of SIBs compared to mild over‐discharge.

**Table 3:** Summary of effects of mild electrical abuse on SIBs safety characteristics.

Analysis method	Results	
	Overcharge	Over‐discharge
	Single electrical abuse	
Capacity	No degradation	No degradation
Cycle temperature	Significant rise	No significant change
Electrochemical characteristic	Sodium inventory increased slightly, SEI film thickens; Deposition of side reaction products increases; Interface passivation intensifies	Slight loss of active material, SEI film dissolved; The reaction rate on the electrode surface decreased
Sodium plating	Increase	Decrease
Thermal runaway behavior	Temperature platform reduction; Trigger time advanced	No significant change
Morphology	Cathode particles crack; Anode plating sodium increase	Cathode particles do not change significantly; Anode plating sodium decrease
Element composition	Cathode consumption of oxygen and sodium containing substances; Promote the deposition of organic by‐products on anode surface	Cathode oxygen and fluorine compounds increased; Anode sodium compound reduce and iron ions dissolve
Material structure	Well maintained	Well maintained
Thermal stability	Cathode material has little effect; Anode material has great influence	Cathode and anode materials have little effect
	Long‐term electrical abuse cycle	
Electrochemical characteristic	Impedance significantly increase; SEI thickening; Significant capacity decline	Impact relatively small
Thermal stability	Sodium plating increase; Cycle temperature increase	Sodium plating decrease; Limited temperature effect

Compared with existing reports on mild electrical abuse in LIBs, there are differences in material behavior, degradation mechanisms, and severity between SIBs and LIBs under abuse conditions. In LIBs, mild overcharge causes lithium plating, while over‐discharge leads to copper plating, both of which reduce thermal stability. Notably, copper plating during over‐discharge has a more significant negative impact on thermal characteristics than lithium plating during overcharge. In contrast, in SIBs, sodium plating during overcharge is the primary factor that reduces thermal stability, advances the thermal runaway trigger point, and increases temperature rise during cycling. Sodium plating dissolution during over‐discharge has relatively lighter effects on safety and performance. Therefore, in practical applications, the risks associated with overcharging in SIBs should be particularly emphasized.

## 5 Conclusion

The impact of mild electrical abuse on SIBs is not caused by the independent failure of a single component but is instead the result of multi‐component synergistic degradation. This study systematically investigates the degradation mechanisms and pathways of SIBs safety characteristics under both single and long‐term electrical abuse cycles. It elucidates the coupling mechanisms between electrode materials, electrochemical signals, and heat generation characteristics, leading to the following conclusions:

This study systematically investigates the effects of mild electrical abuse on the electrochemical performance and thermal stability of NFM SIBs. Advanced electrochemical testing and material characterization techniques are employed to investigate the degradation mechanisms and pathways of safety characteristics in SIBs under both single electrical abuse and long‐term abuse cycling, leading to the following findings:

1. Mild electrical abuse has minimal impact on battery capacity, it significantly alters the interface impedance and performance of active electrode materials. Overcharge increases SEI membrane impedance and intensifies side reactions, whereas over‐discharge leads to SEI membrane dissolution and active material loss.
2. Mild overcharge lowers the TR trigger temperature and accelerates the temperature rise rate due to sodium plating, significantly increasing TR risk. In contrast, over‐discharge minimally affects TR behavior and may slightly delay the temperature rise.
3. Long‐term electrical abuse will significantly degrade the electrochemical performance of the battery. Furthermore, long‐term overcharge will cause sodium plating accumulation on the anode surface, with the extent of plating positively correlated with the number of overcharge cycles.

## Conflict of Interest

The authors declare no conflict of interest.

## Supporting information



Supporting Information

## Data Availability

Research data are not shared.
