# Supplementary material for: Revealing the Hazard of Mild Electrical Abuse on the Safety Characteristics of NaNi1/3Fe1/3Mn1/3O2 Cathode Sodium‐Ion Battery
Source: Adv Sci (Weinh). 2025 Apr 25;12(26):2501649. doi: 10.1002/advs.202501649 (PMC12245037; doi:10.1002/advs.202501649)
Supplement: Supplementary file 1 — Supporting Information [file ADVS-12-2501649-s001.docx]

Supplementary Materials for

**Revealing the Hazard of Mild Electrical Abuse on the Safety Characteristics of NaNi_1/3_Fe_1/3_Mn_1/3_O_2_ Cathode Sodium-Ion Battery**

Qinghua Gui^1^, Bowen Jin^1^, Peng Liu^1^, Kun Yu, Jiarui Zhang^1^, Lei Mao*^1,2^

^1^ Department of Precision Machinery and Precision Instrumentation, University of Science and Technology of China, Hefei, China.

^2^ Institute of Advanced Technology, University of Science and Technology of China, Hefei, China

*Corresponding author. Email: leimao82@ustc.edu.cn (Lei Mao)

**This file includes:**

Figures S1 to S5

Tables S1 to S5


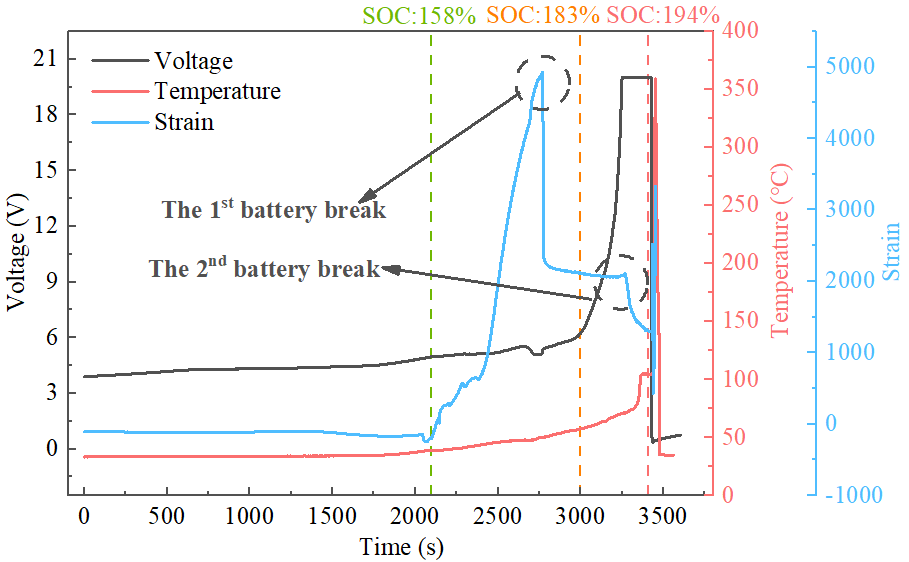


**Figure S1.** Overcharge induced thermal runaway process.


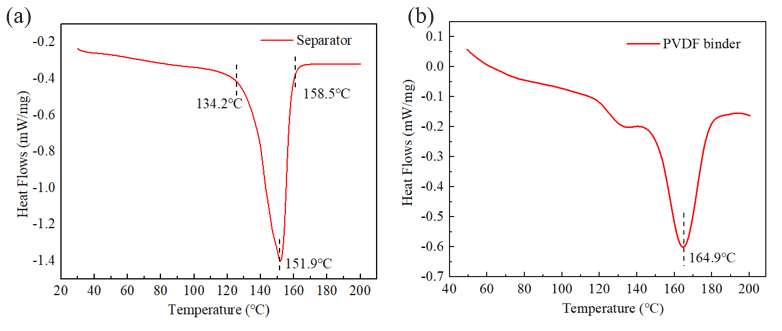
 **Figure S2.** DSC test results. a) separator. b) binder.


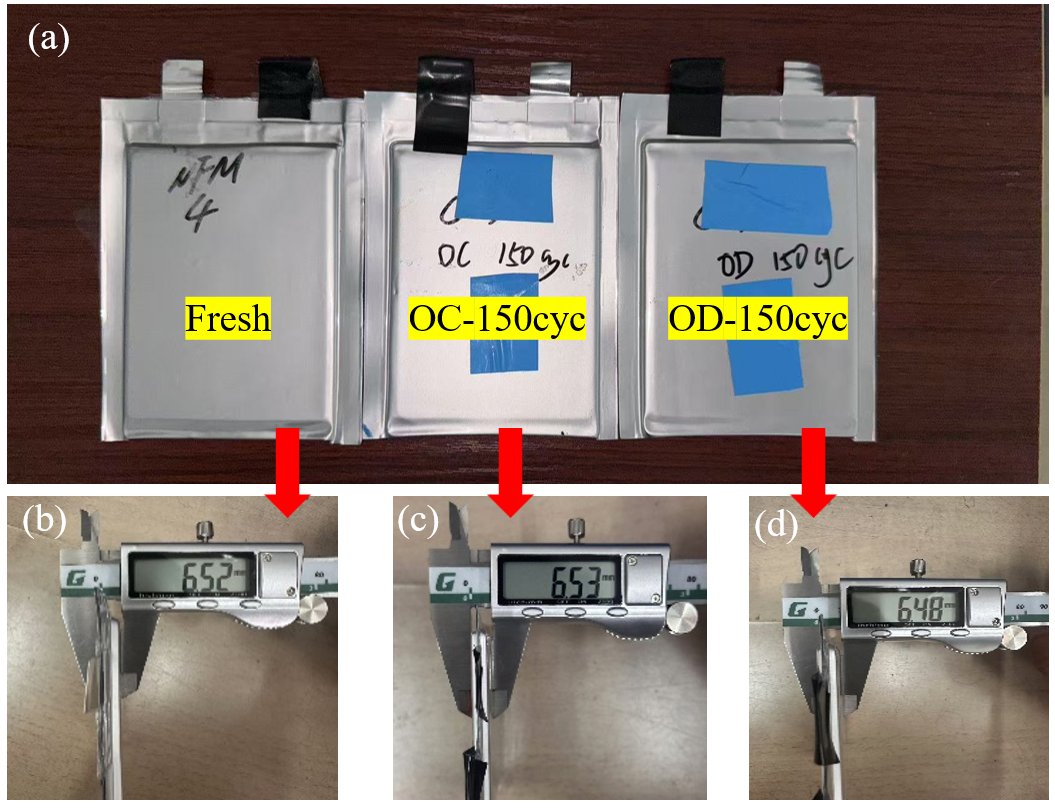


**Figure S3.** Comparison of appearance before and after electrical abuse. a) Top view. b) Fresh battery thickness. c) Overcharge cycle 150 times battery thickness. d) Over-discharge cycle 150 times battery thickness.


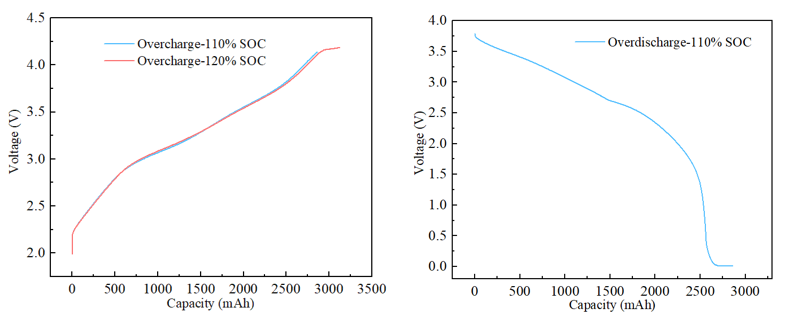


**Figure S4.** Overcharge and over-discharge curves. a) Overcharge. b) Over-discharge.


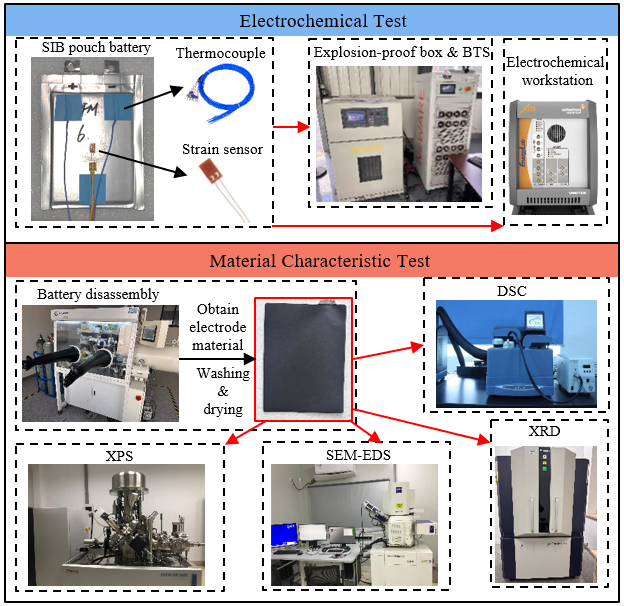


**Figure S5.** Schematic diagram of electrochemical test and material characterization test.

**Table S1.** Detailed ICP quantitative data of cathode.

| Samples | Elements | | | | | |
| --- | --- | --- | --- | --- | --- | --- |
|  | Na (wt%) | Ni (wt%) | Fe (wt%) | Mn (wt%) | O (wt%) | C  (wt%) |
| Fresh | 14.44 | 11.85 | 11.31 | 12.51 | 31.45 | 18.44 |
| Overcharge 110% SOC | 13.89 | 12.55 | 12.02 | 13.06 | 27.81 | 20.67 |
| Overcharge 120% SOC | 11.97 | 12.43 | 12.07 | 13.24 | 25.92 | 24.37 |
| Over-discharge 110%SOC | 13.91 | 11.57 | 13.16 | 13.19 | 23.04 | 25.13 |

**Table S2.** Detailed ICP quantitative data of anode.

| Samples | Elements | | |
| --- | --- | --- | --- |
|  | Na (wt%) | O (wt%) | C (wt%) |
| Fresh | 5.43 | 2.90 | 91.67 |
| Overcharge 110% SOC | 6.40 | 8.27 | 85.33 |
| Overcharge 120% SOC | 7.27 | 9.53 | 83.20 |
| Over-discharge 110%SOC | 5.54 | 6.33 | 88.13 |

**Table S3.** Specifications of NFM/HC sodium-ion battery.

| Specifications | Values |
| --- | --- |
| Rated capacity | 2.6Ah |
| Cutoff voltage | 1.5-3.9V |
| Mass | 67.4g |
| Areal density | 290g/m^2^(NFM)/115 g/m^2^(HC) |
| Compaction density | 3.0 g/cm^3^ (NFM)/0.95 g/cm^3^ (HC) |
| N/P | 1.09 |
| Battery body size | 82×61×6.5mm |
| Electrode thickness | 110 μm/134μm |
| Aluminum foil thickness | 12 μm/13 μm |
| Electrode layer number | 19(NFM)/20(HC) |
| Separator | Single-sided ceramic separator |
| Electrolyte | 1M NaPF6 in EC: PC: DEC: EMC=1:3:1:4 Vol% with 1% FEC |

**Table S4.** SIBs battery consistency test step.

| Number | Step | Value | Cut-off voltage |
| --- | --- | --- | --- |
| 1 | rest | 10 h | / |
| 2 | CD | 0.2 C | 1.5 V |
| 3 | rest | 1 h | / |
| 4 | CC-CV | 0.2 C/0.05 C | 3.9 V |
| 5 | rest | 1h | / |
| Cycle | 2 to 5 (10 cycles) | / | / |

**Table S5.** Electrochemistry and temperature test step.

| No.  Step | 1 | 2 | 3 | 4 | Test item |
| --- | --- | --- | --- | --- | --- |
| rest | 10 h | | | | / |
| CC  &  CD | 100% SOC | 110% SOC | 120% SOC | -110% SOC  &  100% SOC | IC,  EIS |
| rest | 2 hours | | | | DV |
| CD | 1.5 V | | | | / |
| rest | 1 hour | | | | / |
| CC | 3.9 V | | | | Capacity,  Temperature |
